# Molecules Isolated from Mexican Hypoglycemic Plants: A Review

**DOI:** 10.3390/molecules25184145

**Published:** 2020-09-10

**Authors:** Sonia Marlen Escandón-Rivera, Rachel Mata, Adolfo Andrade-Cetto

**Affiliations:** 1 Laboratory of Etnofarmacology, Faculty of Sciences, National Autonomus University of Mexico, Mexico City 4510, Mexico; soniaer@ciencias.unam.mx; 2 Faculty of Chemistry, National Autonomus University of Mexico, Mexico City 4510, Mexico; rachel@unam.mx

**Keywords:** Mexican medicinal plants, hypoglycemic, α-glucosidase inhibitor, insulin sensitizer, insulin secretion

## Abstract

Like in many developing countries, in Mexico, the use of medicinal plants is a common practice. Based on our own field experience, there are at least 800 plants used for treating diabetes nowadays. Thus, their investigation is essential. In this context, this work aims to provide a comprehensive and critical review of the molecules isolated from Mexican hypoglycemic plants, including their source and target tested. In the last few years, some researchers have focused on the study of Mexican hypoglycemic plants. Most works describe the hypoglycemic effect or the mechanism of action of the whole extract, as well as the phytochemical profile of the tested extract. Herein, we analyzed 85 studies encompassing 40 hypoglycemic plants and 86 active compounds belonging to different classes of natural products: 28 flavonoids, 25 aromatic compounds, other than flavonoids, four steroids, 23 terpenoids, 4 oligosaccharides, and 1 polyalcohol. These compounds have shown to inhibit α-glucosidases, increase insulin secretion levels, increase insulin sensitivity, and block hepatic glucose output. Almost half of these molecules are not common metabolites, with a narrow taxonomic distribution, which makes them more interesting as lead molecules. Altogether, this analysis provides a necessary inventory useful for future testing of these active molecules against different hypoglycemic targets, to get a better insight into the already described mechanisms, and overall, to contribute to the knowledge of Mexican medicinal plants.

## 1. Introduction

Diabetes mellitus is a long-term condition that occurs when there are high glucose levels in a person’s blood because their body cannot produce any or enough insulin or cannot effectively use the insulin it produces. Type 2 diabetes (T2D), due to a progressive loss of adequate β-cell insulin secretion frequently on the background of insulin resistance [1], accounts for nearly 90% of cases. The International Diabetes Federation estimated 463.0 million people living with T2D worldwide, Mexico ranks in sixth place, with 12.8 million diabetics [2]. Due to the idiosyncrasy of the Mexican population, normally, type 2 diabetic patients mainly in the rural areas of the country use a combination of medicinal plants and prescribed medicines [3]. In Mexico, at least 300 plants have been reported as hypoglycemics [4] but based on our own field experience [5,6], we estimate that at least 800 plants are used nowadays for treating diabetes.

Medicinal plants have been a resource for healing in local communities around the world. Still, 85% of the world’s population depends on plants for primary healthcare [7]. In addition, they are an important resource for drug discovery, with 80% of all synthetic drugs deriving from them [8]. It is important to remark that medicinal plants can lead us to the discovery of new molecules useful for the development of new drugs for treating T2D and its complications.

Diverse strategies are utilized for control T2D; first, a combination of diet and exercise is in order; then, patients are prescribed oral hypoglycemic agents, initially a single drug, and later, a combination of two or more drugs. Patients can remain with exercise, diet, and oral hypoglycemic agents for a long time, providing they follow a healthy lifestyle. When this does not happen, they receive an oral hypoglycemic, first as monotherapy and then as a combination; if this does not control the blood glucose levels then external insulin is prescribed. The starting point for living well with diabetes is an early diagnosis, the longer a person lives with undiagnosed and untreated diabetes, the worse their health outcomes are likely to be. The World Health Organization (WHO) recommends a series of cost-effective interventions to improve patient outcomes. These interventions include blood glucose control through diet, physical activity, and, if necessary, medication [9]. While the American Diabetes Association (ADA) remarks that lifestyle changes such as dietary modification and increased physical activity can be very effective in improving glycemic control, over the long-term, most individuals with T2D will require medications to achieve glycemic control [10].

Until the beginning of the 1990s, sulfonylureas and metformin were the essential oral hypoglycemic agents available. Since then, new therapeutic agents have appeared in the pharmaceutical market. For example, acarbose, an alfa glucosidase inhibitor (AGI), was launched in Germany in 1990; the thiazolidinediones (TZD) insulin sensitizers, in 1996; exenatide, a glucagon-like peptide 1 (GLP1) receptor agonist, in 2005; sitagliptin, a dipeptidyl peptidase 4 (DPP-4) inhibitor, in 2006; and canagliflozin, a sodium–glucose cotransporter 2 (SGLT-2) inhibitor, in 2013 [11]. All these compounds have targets never described before. Acarbose and exenatide are natural products; other AGIs derive from natural products while metformin and canagliflozin are inspired by natural products. New targets to control hyperglycemia continuously appear, as we understand better the pathophysiology of T2D.

The research of traditional medicine can lead us to the discovery of new hypoglycemic compounds, associated with the traditional use of the plant. From an ethnopharmacological perspective, it is a start line to prove molecules isolated from plants traditionally used to treat T2D, although it is an intrinsically complex task demanding a highly sophisticated multidisciplinary approach.

In the last few years, some researchers have focused on the study of Mexican hypoglycemic plants. Most works describe the hypoglycemic effect or the mechanism of action of the whole extract, as well as the phytochemical profile of the tested extract. In the best of the cases, molecules are isolated from organic extracts but not tested against specific targets.

This work aims to provide a comprehensive and critical review of the molecules isolated from Mexican hypoglycemic plants. The studies analyzed included those reporting the hypoglycemic effect (i.e., any effect useful for potentially treating diabetes, in vivo or in vitro) of Mexican medicinal plants, and which possess at least one compound related to this activity. This review will be useful to construct a database useful for future testing of these molecules against different hypoglycemic targets.

## 2. Results

**Figure 1:**
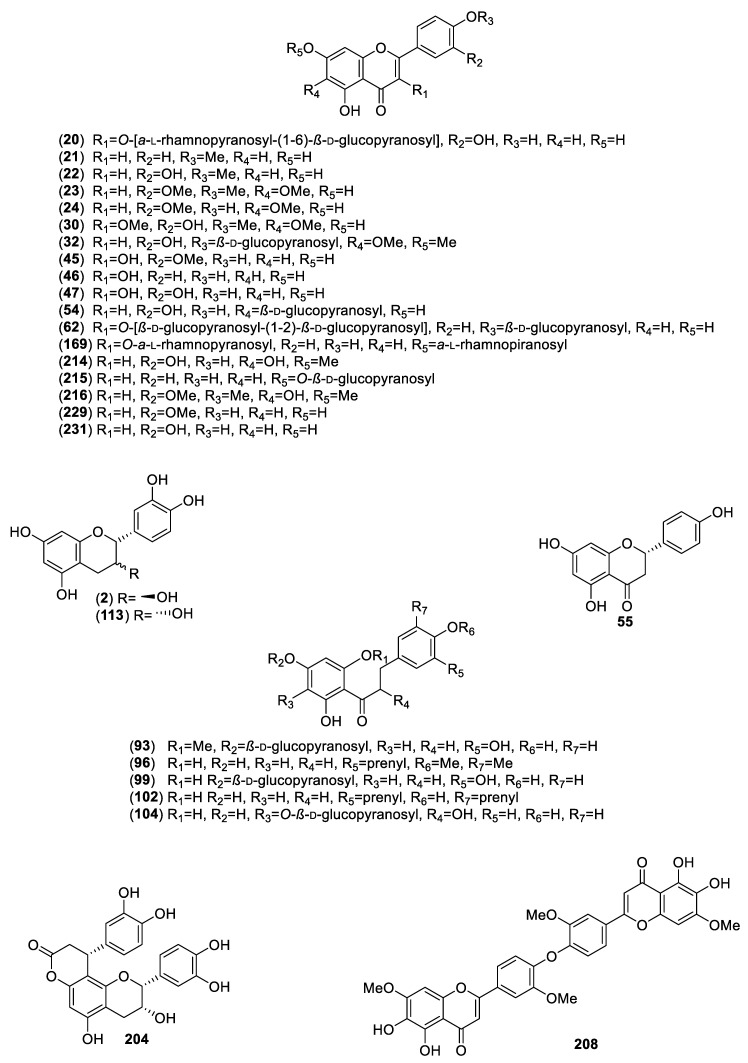
Flavonoids from Mexican plants with hypoglycemic activity.

**Figure 2:**
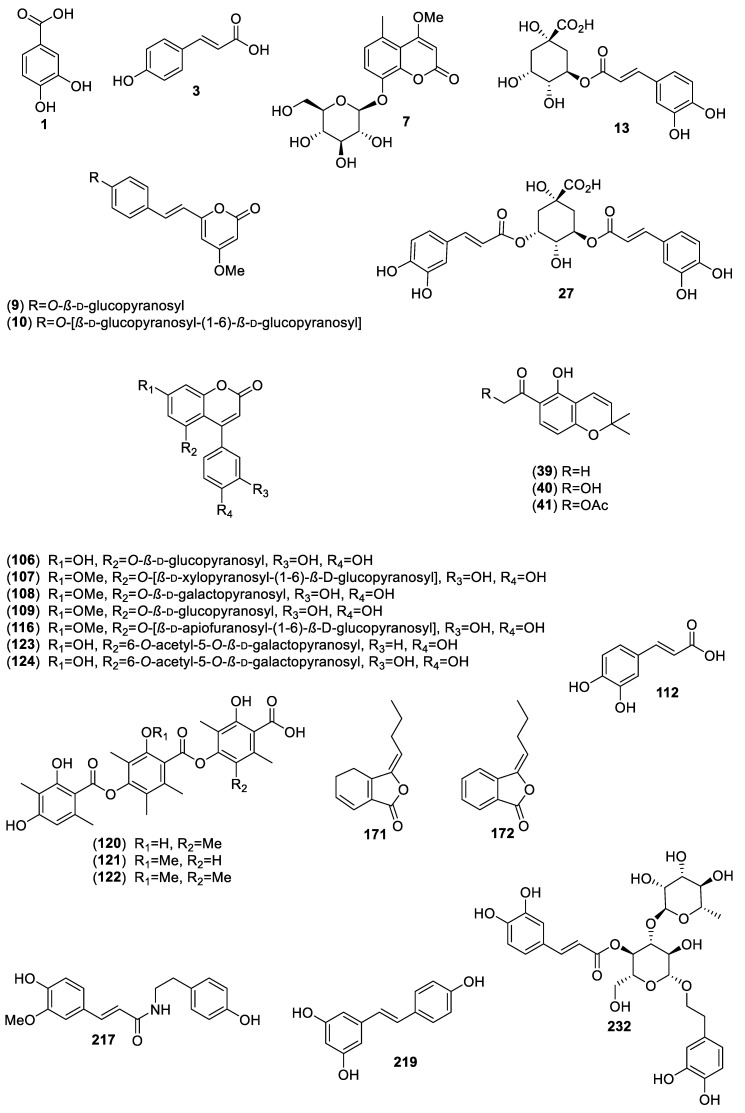
Aromatic compounds, other than flavonoids, from Mexican plants with hypoglycemic activity.

**Figure 3:**
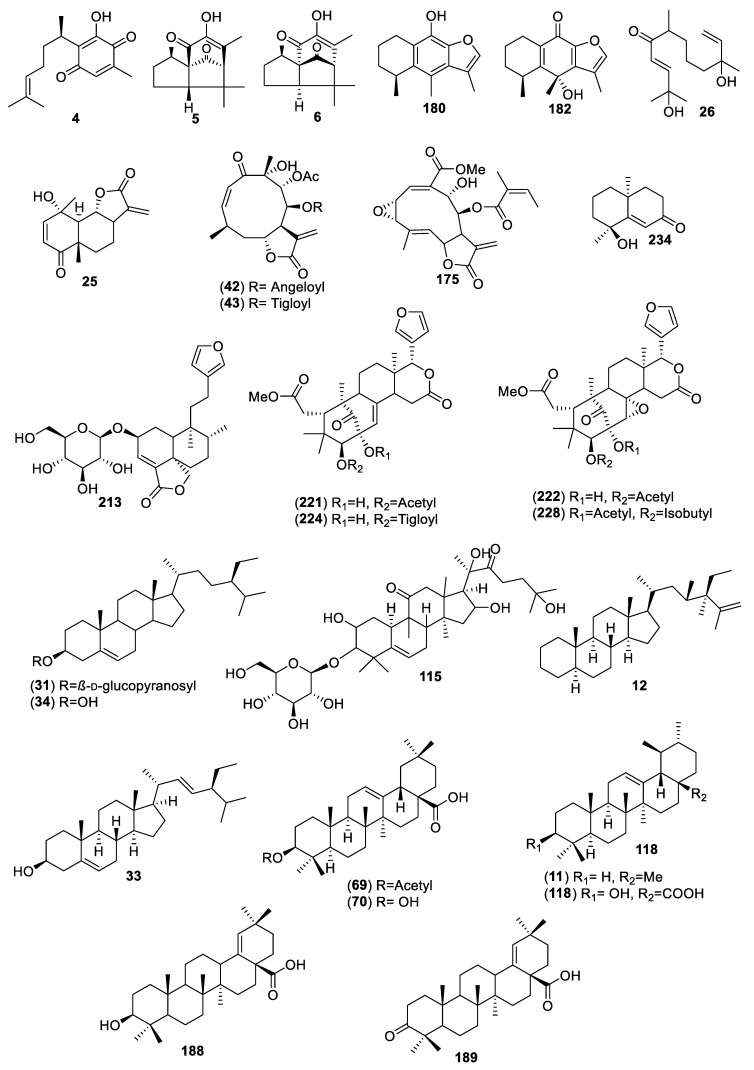
Terpenoids and steroids from Mexican plants with hypoglycemic activity.

**Figure 4:**
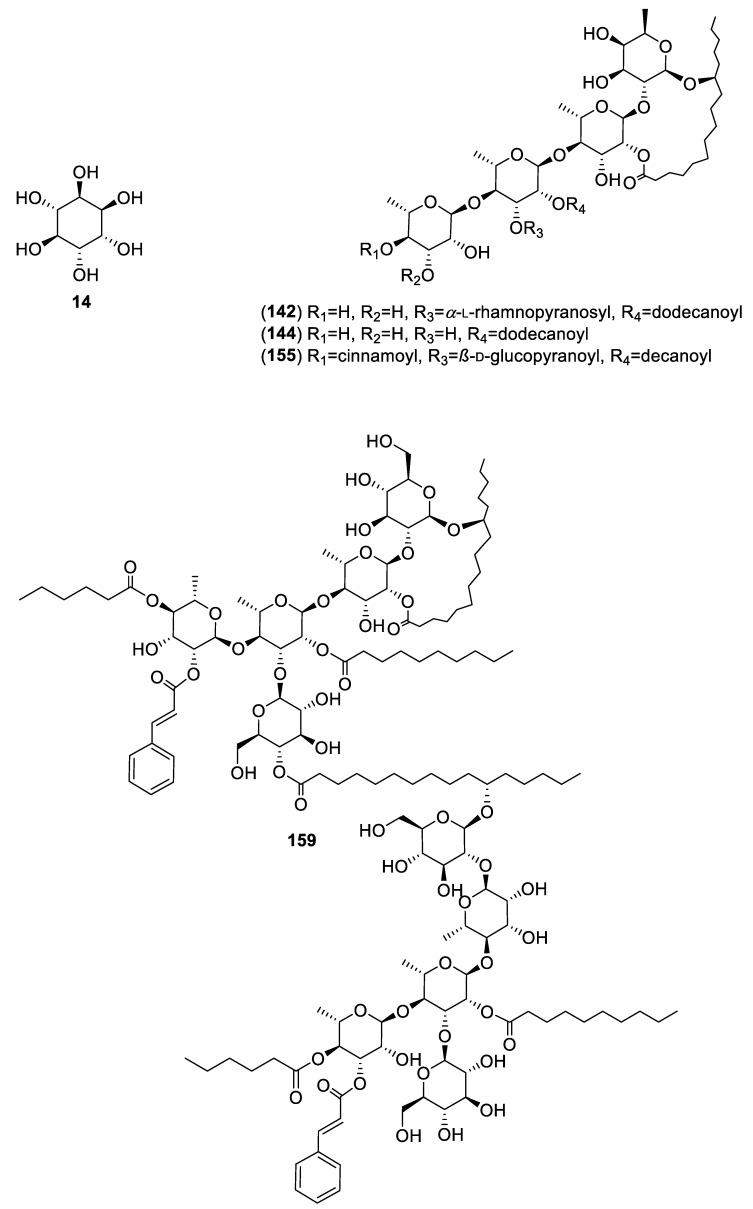
Carbohydrates and a cyclitol from Mexican plants with hypoglycemic activity.

For this review, we selected and analyzed eighty-five works encompassing 40 hypoglycemic plants. Altogether, eighty-six active compounds were found, including 28 flavonoids, 25 aromatic compounds, other than flavonoids, 4 steroids, 23 terpenoids, 4 oligosaccharides, and 1 polyalcohol. Table 1 summarizes their natural sources, and Figure 1, Figure 2, Figure 3 and Figure 4 their corresponding structures. These compounds are representative of the major class metabolites of secondary metabolites from plants.

Among the flavonoids, the most abundant were flavonols (**20**, **30**, **45**–**47**, **62**, **169**), glycosides or not, and flavones (**21**–**24**, **32**, **214**–**216**, **229**, **231**), although a few dihydrochalcones (**96**, **102**, **80**, **93**, **99**, **104**), a biflavone (**208**), a flavanone (**55**), and three flavan-3-ols (**2, 113**, **204**) are included. The aromatic compounds comprise eight coumarins, one simple (**7**), and seven 4-phenylcoumarins (**106**–**109**, **116**, **123** and **124**); six hydroxycinnamic acids (**3**, **13**, **27**, **112**, **217**, **232**); three chromenes (**39**–**41**); three depsides (**120**–**122**); two phthalides (**171** and **172**); two α-pyrone glycosides (**9** and **10**); one stilbene (**219**); and one hydroxybenzoic acid (**1**). The terpenoids included 11 sesquiterpenes (**4**–**6**, **25, 26**, **42**, **43**, **175, 180**, 1**82**, **234**), of which four were sesquiterpene lactones (**25**, **42**, **43**, **175**) of the eudesmanolide, heliangolides, and melampolides types; the remaining were a nor-sesquiterpene (**234**); one acyclic diol (**26),** two eremophylanes (**180**, **182**), and one bisabolene (**4**) and its rearranged products (**5**, **6**). Besides, one neo-clerodane diterpene (**213**); four limonoids (**221**, **222**, **224**, **228**); one cucurbitane (**115**); four oleananes (**69**, **70, 188, 189**); and two ursanes (**11** and **118**) completed the terpenoid family. Among the steroids, (**12**, **31**, **33, 34)**, compound **12** is unusual and has not been reported again from any natural source. The polyalcohol was the important cyclitol **14**, and the oligosaccharides (**142**, **144**, **155**, **159**) belong to the resin glycosides type (Figure 4).

The acute hypoglycemic effect was reported for 61 compounds: 19 Flavonoids (**2, 20, 21**–**24, 30, 32, 45**–**47, 54, 55, 62, 104, 113, 169, 204, 214)**, eight coumarins (**7, 106**–**109, 116, 123, 124**) two pyrones (**9** and **10**), one depside (**122**), two phthalides (**171** and **172**), five phenylpropanoids (**3, 13, 27, 112, 219)**, nine sesquiterpenes (**4**–**6, 42, 43, 25, 180, 182, 234**), two ursanes (**11** and **118**), three limonoids (**221, 222, 224**), one cucurbitane (**115**), four oleananes (**69, 70, 188, 189**) three steroids (**12, 31, 33)** and one ciclytol (**14)**. Eleven compounds displayed chronic hypoglycemic effect: Two flavonoids (**2** and **20**), five phenylcoumarins (**106**–**109, 116)**, one cucurbitane (**115**), and three steroids (**31, 33, 34**).

Forty-four compounds inhibited α-glucosidases, in vivo and/or in vitro: 17 Flavonoids (**2, 20, 45**–**47, 21**–**23, 54, 55, 113, 208, 214**–**216, 229, 23)**, three coumarin (**7, 109, 116**), three chromenes (**39**–**41**), three depsides (**120**–**122**), one phtalide (**172**), four phenylpropanoids (**13, 27, 217, 232**), eight sesquiterpenes (**4**–**6, 26, 42, 43, 175, 234**) including three lactones, one diterpene (**213**) and, four oligosaccharides (**142, 144, 155, 159**). Nineteen compounds increase insulin levels: Eight flavonoids (**2**, **20, 23, 24, 45**–**47, 204**), two phenylpropanoids (**3** and **112**), four sesquiterpenes (**25, 180, 182, 234**), three limonoids (**221, 224, 228**), one steroid (**34**) and one cucurbitane (**115**). Twenty compounds augmented insulin sensitivity: Nine flavonoids (**2, 20, 23, 24, 45**–**47, 113, 169**), two phenylpropanoids (**13** and **112**), one hydroxybenzoic acid (**1**), seven terpenoids (**70, 118, 188, 189, 221, 224, 228**) and one steroid (**34**). Finally, nine compounds inhibit glucose 6-phosphatase activity: three flavonoids (**20, 23, 24**), two phenylpropanoids (**13** and **112**), one hydroxybenzoic acid (**1**), and three limonoids (**221, 224, 228**).

Thirteen compounds of the 86-active isolated from Mexican plants have been tested elsewhere. In most cases, the studies confirm the activity found in Mexico, as in the case of α-glucosidase inhibitors **45**, **47**, **215**, **229**, **231**, and **232**.

Table 2 shows the complete list of compounds, the plant source, types of tests performed, and their mechanism of action. In addition, the activity of the compounds assessed elsewhere in the world is included. This is because, in many works, the mechanism of action of the compounds isolated from Mexican plants relies on the work performed by other authors.

It is important to mention that most compounds listed in Table 1; Table 2 were isolated from organic extracts, and in a few cases from the traditionally aqueous preparation. Only in one case, the active principle of a hypoglycemic essential oil was established [28,29].

From the nine types of pharmacological agents currently used to treat T2D (sulfonylureas, meglitinides, biguanides, thiazolidinediones, alpha glucosidase inhibitors, DPP-4 inhibitors, bile acid sequestrants, dopamine agonists, and sodium–glucose transport protein 2 inhibitors), the compounds obtained from Mexican hypoglycemic plants share one or more of their mechanisms of action. The mechanisms most frequently found were α-glucosidase inhibition, insulin secretion or sensitization, and/or inhibition of hepatic glucose output (HGO). The α-glucosidase inhibitory effect is clear from the assays; the hypoglycemic effect is only throughout the inhibition of the enzyme. In addition, the assays required to demonstrate the activity in vivo or in vitro are easier to perform. When the compounds increase insulin plasmatic levels, the mechanism action, however, is not straightforward because they can target the pancreatic β-cells, increase GLP1 production in the gut, inhibit DPP4, or even act through unknown mechanisms. Those behaving as insulin sensitizers might be affecting different pathways.

### 2.1. Glucosidases Inhibitors

α-Glucosidase is the key enzyme involved in the digestion of carbohydrates; inhibitors of this enzyme can slow down the liberation of absorbable monosaccharides from dietary complex carbohydrates. The action mechanism of AGIs causes a decrease in glucose levels as well as osmotic effects. The disaccharides which are not digested will endure in the intestinal lumen and may lead to side effects such as diarrhea, flatulence, and abdominal pain [122]. AGIs block the enzymatic reaction particularly because of their nitrogen component. Thus, they must be present at the site of enzymatic action at the same time as the carbohydrates. Three AGIs are now in therapeutic use worldwide; acarbose isolated from culture filtrates of *Actinoplanes*, was first described by Schmidt in 1977 and introduced onto the market in 1990; miglitol, a semisynthetic derivative of 1-deoxynojirimycin, from *Bacillus* and *Streptomyces* spp; and voglibose, which is prepared from validamycin A, a product of *Streptomyces hygroscopicus* [84]. In Mexico, only acarbose is available.

While flavonoids and phenylpropanoids are well-known α-glucosidase inhibitors, depsides, phthalides, 4-phenylcoumarins, chromenes, α-pyrones, and sesquiterpene lactones are not. The most important AGIs isolated from Mexican plants are α-pipitzol (**5**), β-pipitzol (**6**), 6-acetyl-5-hydroxy-2,2-dimethyl-*2H*-chromene (**39**), calein A (**42**), calein C (**43**), 3-(*Z*)-butylidenephthalide (**171**), perfoliatin A (**175**), amarisolide (**213**), and pedalitin (**214**), and the 4-phenylcoumarins **106** and **109** (Table 2). This ranking arose because these compounds showed in vivo and in vitro action. Some of them possessed other mechanisms as discussed later. Kinetic analysis indicated that 6-acetyl-5-hydroxy-2,2-dimethyl-*2H*-chromene (**39**) and calein C (**43**) behaved as noncompetititive and mixed (*K*i of 0.13 m) type inhibitors, respectively, against *S. cerevisiae* α-glucosidase [28]. This behavior was consistent with the in silico predictions with AutoDock4, which revealed that **43** bound close to the catalytic site of the enzyme, with a *K*i value of 0.30 μM while compound **39** bound in regions different from the catalytic area, with a *K*i value of 13 μM. 3-(*Z*)-Butylidenephthalide (**171**), was also characterized as noncompetitive inhibitor with a *K*i of 4.86 mM [67]. The 4-phenylcoumarins **106** and **109** were also good inhibitors of yeast α-glucosidase, but their aglycones were most active [115]. Another unusual α-glucosidase inhibitor was the sesquiterpene salvinine (**26**), which behaved as a mixed type of inhibitor and because its size and properties form an interesting molecule for optimization to increase its potency [22]. Perfoliatin (**175**) [68] amarisolide (**213**), pedalitin (**214**) [85] were not subjected to kinetic analysis but in silico analysis predicted that these compounds bind to yeast-α-glucosidase at the same site than acarbose.

### 2.2. Effect over Insulin Secretion

Different types of compounds increase insulin secretion. The first group described were the sulphonylureas, SUs, which stimulate the β-cells of the pancreas to release insulin. SUs binds to the SU receptor (SUR), a subunit of the potassium ATP-dependent (KATP) channels in the β-cell membrane, which eventually blocks the potassium channels and facilitates the influx of Ca^2+^ into the cell, leading to membrane depolarization and subsequently insulin exocytosis. The main side effect is hypoglycemia; in Mexico, the most used SU is glibenclamide [123].

The glinides include drugs such as repaglinide (the only one available in Mexico), which belong to the group of nonsulfonylurea insulin secretagogues, with a rapid onset and reduced interval of action. They act by binding to the SUR1 receptor of the pancreatic β-cells (in a different site than SUs), they have less affinity for the receptor in comparison to SUs. Unfortunately, the glinides produce many adverse effects such as nausea, vomiting, diarrhea, constipation, gastrointestinal upsets, and abdominal pain. Due to their short duration of action, the associated hypoglycemia is much less recurrent than SUs [124].

Incretin-based medicines; after the intake of oral glucose, a phenomenon called incretin effect occurs, which increases insulin secretion up to 50–70%. The incretin hormones, Glucagon-like peptide-1 (GLP-1) and the glucose-dependent insulinotropic polypeptide (GIP) are intestinal peptides released after ingestion of food; they augment the pancreatic islet function by increasing insulin release and suppressing the synthesis of glucagon. The incretin-based medications include two classes of drugs, dipeptidyl peptidase-4 (DPP-4) inhibitors and GLP-1 receptor agonists. Both medications diminished the glycemic abnormalities. The incretin hormones GLP-1 and GIP inhibited gastric emptying, decreased appetite and food intake, and reduced glucagon’s secretion. The life span of GLP-1 is very short, as DPP-4 converts GLP-1 into an inactive form. While DPP-4 inhibitors increase the life span of the incretin hormones, the GLP1 agonists imitate the glucoregulatory actions of GLP-1 and diminish the release of glucagon, thus improving the glucose-dependent secretion of insulin. At the same time, common symptoms associated with the use of GLP-1 receptor agonists include gastrointestinal symptoms, mainly nausea and vomiting, headaches, reactions at the site of injection, and nasopharyngitis [125,126]. In Mexico, exenatide (a GLP1 agonist) is available as well as the DPP-4 inhibitor (sitagliptin).

Based on the reported experiments, a few molecules isolated from Mexican plants can increase insulin levels; the most common were again phenylpropanoids and flavonoids. The first group includes the phenylpropanoids *ρ*-coumaric acid (**3**) and caffeic acid (**112**). The relevant flavonoids were eupatilin (**23**) and jaceosidin (**24**) which increased insulin secretion with no specific mechanism; but with the involvement of K^+^-ATP sensitive and Ca^2+^-voltage-dependent channels, which play an important role in insulin secretion [21,22]. The flavonol kaempferol (**46**) restores PA-induced loss of β-cell mass and function through AMPK/mTOR-mediated autophagy. Finally, cinchonain 1b (**204**) induces insulin secretion in vitro and in vivo.

More unusual were 3-*O*-β-d-glucopyranosyl-23,24-dihydrocucurbitacin F (**115**), 5-*O*-d-glucopyranosyl-7-3′,4′-triihydroxy-4-phenylcoumarin (**109**), 5-*O*-[β-d-apiofuranosyl-(1→6)-β-d-glucopyranosyl]-7-methoxy-3′, 4′-dihydroxy-4-phenyl-coumarin (**116**) and other 4-phenylcoumarins, which increase insulin secretion in rats [55]. Compounds **109** and **116** stimulated AMPK phosphorylation in vitro in C2C12 myoblast cells. Thus, these compounds activate the AMPK pathway, which, in turn, could stimulate insulin secretion [127]. Based on these structures, Wang and collaborators in 2017 [128] synthesized a few substituted 4-phenylcoumarins derivatives analogs of compound **109**; their pharmacological investigation showed that those 4-phenylcoumarins possessing adjacent 7,8-dihydroxy groups were equipotent to the standard drug glibenclamide as hypoglycemic agents in vivo. In addition, these compounds inhibited aldose reductase and advanced glycation end-product formation. Altogether, this shows the potential of 4-phenylcoumarins for the development of therapeutic agents for treating diabetes, and how products discovered during the investigation of Mexican hypoglycemic plants are useful as templates for the development of more active compounds [128]. The sesquiterpene lactone arglanin (**25**) was investigated as a hypoglycemic agent in Mexico for the first time. Its nonspecific mechanisms also involved K^+^-ATP sensitive and Ca^2+^-voltage-dependent channels [22]. Finally, the cyclitol l-*chiro*-inositol (**14)**, is also a well-known hypoglycemic agent. Inositol derivatives (among them compound **14**), have been reported to help the action of insulin stimulating glucose uptake in skeletal muscle cells, so they also work as insulin sensitizers [18,129].

### 2.3. Insulin Sensitizers

As mentioned above, T2D has two main components, insulin resistance and a progressive failure in insulin secretion. Insulin Resistance (IR) leads to a loss of response from the peripheral insulin target tissues (mainly the liver, adipose, and muscle tissues) to insulin. IR impairs the ability of the pancreatic cells to synthesize and secrete enough insulin to address the metabolic needs of the body. Insulin sensitizers help target cells in liver, skeletal muscle, and adipose tissue to respond properly to insulin, resulting in their ability to efficiently uptake and metabolize glucose [130].

Glitazones (TZDs) are insulin sensitizers used for treating T2D. TZDs are selective agonists of PPARγ, a transcription factor involved in the regulation of the expression of specific genes present in fat cells and other tissues. TZDs primarily act in adipose tissue where expression of PPARγ predominates. They interfere with the expression and release of mediators of IR (e.g., free fatty acids, adipocytokines, resistin, adiponectin) that originate in adipose tissue, thus resulting in improvement of insulin sensitivity in muscle and liver [131]. Treatment with TZDs might produce adverse hepatic, cardiovascular, osteological, and carcinogenic events [132]; therefore, TZDs were withdrawn for the market in several European countries; in Mexico, rosiglitazone is still available. Because of the importance of insulin sensitizers in the treatment of T2D, new-generation insulin sensitizers classified as direct PPARγ agonists, selective PPARγ modulators, and PPARγ-sparing compounds are in development [130].

Some important molecules acting as insulin sensitizers, but with an unknown mechanism, isolated from Mexican plants are: Protocatechuic acid (**1**), catechin (**2**) oleanolic acid (**70**), and epicatechin (**113**), for these molecules, the HOMA has been calculated. Acacetin (**21**) and diosmetin (**22**) are PPAR agonists. Isorhamnetin (**45**) and quercetin (**47**) increase glucose uptake in skeletal muscle by activation of the JAK/STAT pathway, and CaMKKβ/AMPK and insulin signaling pathways, respectively. Kaempferitrin (**169**) inhibits GLUT4 mediated glucose uptake in differentiated 3T3-L1 cells by interfering with the insulin signaling pathway and by directly interacting with membrane GLUT4. β-sitosterol (**34**) attenuates the insulin receptor substrate-1 serine phosphorylation. However, it up-regulates RNA expression of IR and postreceptor insulin signaling molecules such as IRS-1, β-arrestin 2, Akt, AS160, and GLUT4 with a concomitant increase in the levels of IRS-1, p-IRS1-1, Akt, p-Akt, AS160, and p-AS160 compared with type-2 diabetic rats [118]. Finally, 5-*O*-[β-d-apiofuranosyl-(1→6)-β-d-glucopyranosyl]-7-methoxy-3′,4′-dihydroxy-4-phenyl-coumarin (**116**) stimulated ^3^H-deoxyglucose uptake in C2C12 myoblasts cells after 18 h of incubation. The effect was like metformin and insulin and was concentration-dependent [59].

### 2.4. Inhibitors of Hepatic Glucose Output (HGO)

One important action of insulin is the signaling over the liver when there is not enough insulin or there is some degree of insulin resistance in the hepatic cell, the result is an augmentation of glucose output. The liver plays a crucial role in maintaining blood glucose levels during fasting by converting its stored glycogen to glucose (glycogenolysis) and by synthesizing glucose, mainly from pyruvate and amino acids (gluconeogenesis) [133].

In T2D subjects with mild to moderate fasting hyperglycemia (140–200 mg/dL), basal hepatic glucose production increases by ~0.5 mg/kg min. Consequently, during the overnight sleeping hours (22.00 to 8.00 h), the liver of an 80 kg diabetic individual with modest fasting hyperglycemia adds 35 g of glucose to the systemic circulation. In T2D, insulin resistance in the liver reflects hyperinsulinemia’s failure to suppress gluconeogenesis, resulting in fasting hyperglycemia and decreased liver glycogen storage in the postprandial state. Increased hepatic glucose production occurs early during diabetes, although the increase is more obvious after the onset of insulin secretory abnormalities and insulin resistance in skeletal muscle. T2D is characterized by impaired glucose homeostasis partly due to abnormally elevated HGO [134].

Metformin, the main insulin sensitizer in use worldwide, acts by different mechanisms, diminishing the intestinal glucose absorption, increasing peripheral uptake and use of glucose, and lowering the hepatic synthesis of glucose [122]. Although metformin is an insulin sensitizer, its action over the HGO is also significant; metformin induces mild energy stress in the liver, leading to an increase in AMP concentration that allosterically inhibits the enzyme fructose-1,6-bisphosphatase-(FBP1) to lower HGO. This is a very important mechanism, as the subsequent increase in F-1,6-P2, will activate PK and increase glycolytic flux. Because AMP-inhibited FBP1, a rate-controlling enzyme in gluconeogenesis, it functions as a major contributor to the therapeutic action of metformin [135].

Although in the international literature, polyphenols like kaempferol or caffeic acid have been related to the blockage of the enzyme G6Pase, from Mexican plants, chlorogenic acid a well-characterized inhibitor of the G6Pase T1 translocase system [19]; thus, it can block HGO, has been reported. *ρ*-Coumaric acid (**3**) inhibits fructose-1,6-bisphosphatase, which also leads to the block of HGO; however, this mechanism has not been explored in molecules isolated from Mexican plants.

The limonoids 2-hydroxy-destigloyl-6-deoxyswietenine acetate (**221),** methyl-2-hydroxy-3-β-tigloyloxy-1-oxomeliac-8(30)-enate (**223**), and humilinolide H (**228**), isolated from *S. humilis*, inhibit also glucose-6-phosphatase, representing new leads for further exploration as HGO, agents. The complexity of their structure makes them good candidates for fragment-based drug discovery.

## 3. Discussion

T2D is an important health problem in many countries; Mexico is not an exception. Like in many developing countries, in Mexico the use of medicinal plants is a common practice, for these two main reasons, plants play an essential role in how the Mexican population treats T2D, this situation is beside the institutional medical attention. It is important to understand how these plants produce a hypoglycemic effect for future isolation of promissory molecules for the development of new hypoglycemic agents.

In the present manuscript, we document 86 molecules isolated from Mexican plants, intending to provide a necessary inventory for further studies. Almost half of these molecules are not common metabolites, with a narrow taxonomic distribution, which makes them more interesting as lead molecules. Some of the most complex molecules (i.e., the limonoids, cucurbitans, or the oligosaccharides) are good candidates for fragment-based drug discovery. The studies summarized in Table 1 and Table 2 along with chemical informatics methods and network pharmacology provides useful information for the development of new phytopharmaceutical preparations. In addition, because some of these molecules are present in plants worldwide, Table 2 will be a useful reference for promoting the testing of these compounds in other targets, known or news, and a better insight into the already described mechanisms.

When hypoglycemic plants are studied it is imperative to set the goal of the study; one point is to prove if the plant has a hypoglycemic effect, which can explain the traditional use but another point is to escalate the study to produce a phytomedicine or a single isolated compound, like metformin. The fact that a plant possesses a hypoglycemic effect does not necessarily mean that it will be of clinical use; for this reason, it is essential to study single molecules in known and new pharmacological targets, aiming to have novel hypoglycemic agents of clinical relevance.

In recent years, the investigation of medicinal plants used to treat diabetes in Mexico has improved. However, we still need to achieve the final goal, contributing with a hypoglycemic agent or phytomedicines of significant clinical efficacy; we are just in the initial steps documenting hypoglycemic plants, and progressing in preclinical testing. The works have been narrowed in scope, both from the pharmacological and chemical point of view. The number and diversity of small molecules to address all the different binding sites in the complex biology of diabetes are still awaiting to be isolated from the plants in Table 1, and other plants yet to be discovered. The essential oils of these plants, plenty of small molecules suitable for the development of hypoglycemic agents remain unexplored. The constituents of the essential oils offer good prospects for drug discovery. These constituents meet most conditions required for good drug candidates and offer attractive opportunities for lead optimization or even fragment-based drug discovery. New technologies such as metabolomics can accelerate dereplication and identification of compounds with novel skeletons; routine incorporation of protein–metabolite interaction and cell-based assays, which require small amounts of samples are essential.

Most compounds in Table 1 require testing as glucose reabsorption inhibitors, incretin analogs, DDP4 blockers, bile acid sequestrants, or dopamine agonists. The effect of these molecules over gut microbiota also needs attention since these bacteria play an important role in the control of T2D. We consider essential to test these molecules as glucose reabsorption inhibitors in the kidney because many plants traditionally used for treating T2D are also employed to treat “Mal de Orin”, an entity that people detect when you have urination problems; this type of plants augment the urine flux. The inhibition of hepatic glucose output is also an important target to assess. From there compounds with similar actions like metformin could upsurge.

A single molecule can have more than one mechanism of action. This might be targeting different proteins within the same (network pharmacology) or different (polypharmacology) signaling pathways In the context, of a botanical mixture, a pool of molecules, perhaps in low concentrations, acting by biochemical synergism can lead to the desired effect, hypoglycemic action, without the presence of a major highly bioactive compound. This has been the classic paradigm of phytotherapy. The advantages of dealing with polyvalent drugs are a decrease of drug-drug interactions, less cost for the patient to bear, increased adherence due to lower side effects, the possibility of treating concomitant diseases, just to mention a few. In this review, we found some examples of compounds possessing more than one mechanism. Thus, protocatechuic acid (**1**) is an insulin sensitizer and secretagogue. Quercetin (**47**) is an insulin sensitizer and secretagogue, as well as the inhibitor of α-glucosidases. Epicatechin (**113**) inhibits α-glucosidase and behaves as an insulin sensitizer. Kaempferol (**46**) inhibits hepatic gluconeogenesis, is an insulin sensitizer and inhibits α-glucosidases. Caffeic acid (**112**) induces insulin secretion, decreases hepatic glucose output, inhibits alpha glucosidases, and is an insulin sensitizer. Furthermore, 2-Hydroxy-destigloyl-6-deoxyswietenine acetate (**221**) stimulates insulin secretion due to a partial blockade of K^+^-ATP channels, and a serotonergic modulation on 5-HT2 receptors inhibited hepatic glucose-6-phosphatase, and stimulated muscle glucose uptake and basal oxygen consumption in muscle cells [91]. The 4-phenylcoumaris affects glucose absorption, insulin secretion, and glucose utilization. With these examples, we want to remark the importance of testing 86 described compounds over different action mechanisms.

## 4. Materials and Methods

This study was performed based on the Preferred Reporting Items for Systematic Review and Meta-Analysis (PRISMA) [136]. To obtain pertinent literature, without author bias, peer-reviewed research articles were obtained from the following databases: PubMed, MEDLINE, EMBASE, SCOPUS, SciFinder, and Clarivate Analytics. Inclusion criteria were studies reporting the hypoglycemic effect of Mexican medicinal plants, and at least one pure compound was isolated and selected.

## 5. Conclusions

The use of medicinal plants to manage diabetes is an overwhelming reality that requires being set into the correct health care perspective. Indeed, these plants will keep providing promissory agents for the development of new antidiabetic drugs or phytotherapeutic products. In this review, we summarize new and known hypoglycemic agents from the reported Mexican hypoglycemic plants. These compounds represent only a small fraction of the chemical space of Mexican Medicinal plants. Some of the compounds’ scaffolds are common, in particular those of the phenylpropanoids and flavonoids. Among the terpenoids and steroids, the ursanes and stigmastanes are widespread in nature, while the remaining terpenoids are less common and restricted to some genus of some family plants. Finally, the other aromatic compounds and the resin glycosides are also limited to a few vegetal species. Except for the flavonoids and phenylpropanoids, the hypoglycemic properties and/or the chemical structures of most compounds were discovered in Mexico, following an ethnopharmacological hypothesis. Their mechanisms of actions involved inhibition of α-glucosidase, insulin secretion or sensitization, and/or inhibition of hepatic glucose output. Some of the less common compounds are multitarget which makes them attractive for drug development.

## Figures and Tables

**Table 1:** Isolated Compounds from Hypoglycemic Mexican plants.

Plant/Family/Part	Isolated Compounds	Ref.
*Acacia angustissima* (Mill.) Kuntze (Fabaceae)/Pods.	Protocatechuic acid (**1**); catechin (**2**); *ρ*-coumaric acid (**3**).	[12]
*Acourtia thurberi* (A.Gray) Reveal & R.M.King (Asteraceae)/Roots.	Perezone (**4**); α-pipitzol (**5**); β-pipitzol (**6**); 8-β-d-glucopyranosyloxy-4-methoxy-5-methyl-coumarin (**7**).	[13]
*Acosmium panamense* (Benth.) Yacolev (Fabaceae)/Roots.	Desmethylyangonine (**8**); desmethylangonine-*O*-β-d-glucopyranoside (**9**); desmethylangonine-*O*-β-d-glucopyranosyl-(1→6)-*O*-β-d-glucopyranoside (**10**).	[14,15]
*Agarista mexicana* (Hemsl.) Judd. (Ericaceae)/Bark.	12-Ursene (**11**); 23,24-dimethyl-24-ethyl-stigmast-25-ene (**12**).	[16,17]
*Ageratina petiolaris* (Moc. & Sessé ex DC.) R. M. King & H. Rob (Asteraceae)/Aerial parts.	Chlorogenic acid (**13**); L-*chiro*-inositol (**14)**; 2α-*iso*-valeroyloxyeperuic acid (**15**); benzyl-2-hydroxy-6-methoxybenzoate (**16**); benzyl-2,6-dimethoxybenzoate (**17**); 3-methoxybenzyl 2,6-dimethoxybenzoate (**18**); benzyl-2-hydroxy-3,6-dimethoxybenzoate (**19**).	[18,19]
*Annona cherimola* Mill (Annonaceae)/Leaves.	Rutin (**20**).	[20]
*Anoda cristata (L.)* Schltdl. (Malvaceae)/Aerial.	Acacetin (**21**); diosmetin (**22**).	[21]
*Artemisia ludoviciana* Nutt (Asteraceae)/Aerial.	Eupatilin (**23**); jaceosidin (**24**); arglanin (**25**); salvinine (**26**); 3,5-*di*-*O*-caffeoylquinic acid (**27**).	[22]
*Arracacia tolucensis* (Kunth) Hemsl. (Apiaceae)/Aerial.	(*S*)-(+)-4′-*O*-angeloylvisamminol (**28**); praeruptorin A (**29**).	[23]
*Brickellia veronicaefolia* (Khunt) Gray (Asteraceae)/Aerial.	5,7,3′-Trihydroxy-3,6,4′-trimethoxyflavone (**30**).	[24,25]
*Bromelia karatas* (L) (Bromeliaceae)/Aerial.	β-Sitosterol-3-*O*-β-d-glucopyranoside (**31**); *ρ*-coumaric acid (**3**); cirsiliol-4′-*O*-β-d-glucopyranoside (**32**); stigmasterol (**33**); β-sitosterol (**34**); 1-*O*-feruloyl-3-*O*-*ρ*-coumaroylglycerol (**35**); β-d-(1-*O*-acetyl-3,6-*O*-*trans*-diferuloyl)-fructofuranosyl-α-d-2′,4′,6′-*O*-triacetyl-glucopyranoside (**36**); 1-*O*-*ρ*-coumaroyl-3-*O*-caffeoylglycerol (**37**); 2-propyl-β-d-glucopyranoside (**38**).	[19,26,27]
*Calea oliveri* B.L.Rob. & Greenm (Asteraceae)/Aerial.	6-Acetyl-5-hydroxy-2,2-dimethyl-*2H*-chromene (**39**); 6-hydroxyacetyl-5-hydroxy-2,2-dimethyl-*2H*-chromene (**40**); 6-acetyl-5-hydroxy-2-methyl-2-hydroxymethyl-*2H*-chromene (**41**); caleins A (**42**) and C (**43**); genkwanin (**44**); isorhamnetin (**45**); kaempferol (**46**); quercetin (**47**); herniarin (**48**); scoparone (**49**); 4′,7-dimethylapigenin (**50**); curcumene (**51**); spathulenol (**52**); caryophyllene oxide (**53**). acacetin (**21**); 3,5-*di*-*O*-caffeoylquinic acid (**27**).	[28,29]
*Cecropia obtusifolia* Bertol. (Urticaceae)/Leaves.	Chlorogenic acid (**13**); isoorientin (**54**).	[30,31,32,33]
*Cochlospermum vitifolium* (Willd) (Bixaceae)/Bark.	(±)-Naringenin (**55**).	[34,35]
*Coriandrum sativum L.* (Apiaceae)/Aerial.	Rutin (**20**).	[36]
*Cucurbita ficifolia Bouché* (Cucurbitaceae)/Fresh mature and immature fruits.	*ρ*-Coumaric acid (**3**); stigmast-7,22-dien-3-ol (**56**); salicin (**57**); stigmast-7-en-3-ol (**58**); *ρ*-hydroxybenzoic acid (**59**).	[37,38,39,40,41]
*Equisetum myriochaetum* Schlecht. & Cham. (Equisetaceae)/Aerial.	Kaempferol-3-*O*-sophoroside (**60**); kaempferol-3,7-*di*-*O*-β-d-glucopyranoside (**61**); kaempferol-3-*O*-sophoroside-4′-*O*-β-d-glucopyranoside (**62**); caffeoyl-methylate-4-β-d-glucopyranoside (**63**).	[19,32,42,43,44]
*Eysenhardtia platycarpa* Pennell & Saff. (Fabaceae)/Leaves, branches and bark.	(1″*R*)-5,4′,1″-Trihydroxy- 6,7-(3″,3″-dimethyl chroman) flavone (**64**); 5,7-dihydroxy-6-methyl-8-prenyl flavanone (**65**); 5,7-dihydroxy-8-methyl-6-prenyl flavanone (**66**); 5,7-dihydroxy-6-prenylflavanone (**67**); 5,7-dihydroxy-8-prenylflavanone (**68**); 3-*O*-acetyloleanolic acid (**69**); oleanolic acid (**70**); 3β-acetoxy-11α,12α-epoxy-oleanan-28,13β-olide (**71**); lupeol (**72**); betulinic acid (**73**); β-sitosterol (**34**); β-sitosterol-3-*O*-β-d-glucopyranoside (**31**); β-sitosteryl palmitate (**74**); 3-*O*-methyl-*myo*-inositol (**75**); (+)-catechin (**2**); (2*S*)-4′-*O*-methyl-6-methyl-8-prenyl-naringenin (**76**); 3,4,6,4′-*O*-methyl-8-prenylnaringenin (**77**); 5-hydroxy-7-methoxy-8-prenyl-flavanone (**78**); (+)-catechin 3-*O*-β-d-galactopyranoside (**79**).	[45,46]
*Eysenhardtia polystachya* (Ortega) Sarg. (Fabaceae)/Leaves and bark.	2′,4′-Dihydroxychalcone-6′-*O*-β-d-glucopyranoside (**80**); α,3,2′,4′-tetrahydroxy-4-methoxydihydrochalcone-3′-*C*-β-d-glucopyranosyl-6′-*O*-β-d-glucopyranoside (**81**); 7-hydroxy-5,8′-dimethoxy-6′α-l-rhamnopyranosyl-8-(3-phenyl-trans-acryloyl)-1-benzopyran-2-one (**82**); 6′,7-dihydroxy-5,8-dimethoxy-8(3-phenyl-trans-acryloyl)-1-benzopyran-2-one (**83**); 9-hydroxy-3,8-dimethoxy-4-prenyl-pterocarpan (**84**); α,4,4′-trihydroxydihydro-chalcone-2′-*O*-β-d-glucopyranoside (**85**); 5,4′-dihydroxy-7,2′-dimethoxylisoflavone (**86**); (*3R*)-5,7-2′,4′-tetrahydroxyl-3′-methoxylisoflavanone (**87**); flemichapparin C (**88**); *neo*-hesperidin dihydrochalcone (**89**); hesperetin dihydrochalcone-glucoside (**90**); aspalathin (**91**); sandwicensis (**92**); 6′-methoxy-sieboldin (**93**); 2′-*O*-α-l-rhamnopyranosyl-α,6′-dihydroxy-4′-acetyl-4-methoxydihydro-chalcone (**94**); 2′-*O*-β-d-glucopyranosyl-4′-methoxy-4-hydroxy-3-isoprenyldihydrochalcone (**95**); 2′,4′,6′-trihydroxy-4,5-dimethoxy-3-isoprenyldihydrochalcone (**96**); 3′-*C*-β-glucopyranosyl-α,2′,4′,6′-trihydroxy-4-methoxy-dihydrochalcone (**97**); 3′-*C*-β-glucopyranosyl-α,2′,4-trihydroxy-4′,6′-dimethoxy-dihydrochalcone (**98**); 3-hydroxyphloretin-4′-*O*-β-d-glucopyranoside (**99**); 3,4′-dihydroxy-2,4,6-trimethoxy-dihydrochalcone (**100**); 2′,4′,4- trihydroxy-3′-methoxy-dihydrochalcone (**101);** 2′,4′,6′,4-tetrahydroxy-3,5-diisoprenyldihydrochalcone (**102**); 3′-*C*-β-glucopyranosyl-α,2′,4′,3,4-pentahydroxydihydroxychalcone (**103**); 3′-*O*-β-d-glucopyranosyl-α,4,2′,4,6′-pentahydroxy-dihydrochalcone (**104**).	[47,48,49,50,51]
*Exostema caribaeum* (Jacq.) Schult. (Rubiaceae)/Stem bark.	Chlorogenic acid (**13**); 5-*O*-[β-d-xylopyranosyl-(1→6)-β-d-glucopyranosyl]-7,3′,4′-trihydroxy-4-phenylcoumarin (**105**); 5-*O*-β-d-glucopyranosyl-7,3′,4′-trihydroxy-4-phenylcoumarin (**106**); 5-*O*-[β-d-xylopyranosyl-(1→6)-β-d-glucopyranosyl]-7-methoxy-3′,4′-dihydroxy-4-phenylcoumarin (**107**); 5-*O*-β-d-galactopyranosyl-7-methoxy-3′,4′-dihydroxy-4-phenylcoumarin (**108**); 5-*O*-β-d-glucopyranosyl-7-methoxy-3′,4′-dihydroxy-4-phenylcoumarin (**109**); 5-*O*-(6′-acetyl-β-d-glucopyranosyl)-7,3′,4′-trihydroxy-4-phenylcoumarin (**110**); 5-*O*-(6′-acetyl-β-d-galactopyranosyl)-7-methoxy-3′,4′-dihydroxy-4-phenylcoumarin (**111**).	[52]
*Hamelia patens* Jacq. (Rubiaceae)/Aerial parts and leaves.	Not isolated but identified chlorogenic acid (**13**); quercetin (**47**); caffeic acid (**112**); epicatechin (**113**); catechin (**2**).	[53,54]
*Hintonia latiflora* (Sessé & Moc. ex DC.) Bullock (Rubiaceae)/Stem bark and leaves/endophytic fungus	5-*O*-β-d-Glucopyranosyl-7,3′,4′-trihydroxy-4-phenylcoumarin (**106**); 5-*O*-[β-d-xylopyranosyl-(1→6)-β-d-glucopyranosyl]-7-methoxy-3′,4′-dihydroxy-4-phenylcoumarin (**107**); 5-*O*-β-d-galactopyranosyl-7-methoxy-3′,4′-dihydroxy-4-phenylcoumarin (**108**); 5-*O*-β-d-glucopyranosyl-7-methoxy-3′,4′-dihydroxy-4-phenylcoumarin (**109**); 25-*O*-acetyl-3-*O*-β-d-glucopyranosyl-23,24-dihydrocucurbitacin F (**114**); 3-*O*-β-d-glucopyranosyl-23,24-dihydrocucurbitacin F (**115**); 5-*O*-[β-d-apiofuranosyl-(1→6)-β-d-glucopyranosyl]-7-methoxy-3′,4′-dihydroxy-4-phenylcoumarin (**116**); desoxycordifolinic acid (**117**); ursolic acid (**118**); 5-*O*-[β-d-xylopyranosyl-(1→6)-β-d-glucopyranosyl]-7,4′-dimethoxy-4-phenylcoumarin (**119**); chlorogenic acid (**13**); thielavins A (**120**), J (**121**) and K (**122**).	[55,56,57,58]
*Hintonia standleyana* Bullock (Rubiaceae)/Stem bark and leaves. This taxon is a synonym of; Hintonia latiflora (Sessé & Moc. ex DC.)	25-*O*-Acetyl-3-*O*-β-d-glucopyranosyl-23,24-dihydrocucurbitacin F (**114**); 3-*O*-β-d-glucopyranosyl-23,24-dihydrocucurbitacin F (**115**); 5-*O*-[β-d-apiofuranosyl-(1→6)-β-d-glucopyranosyl]-7-methoxy-3′,4′-dihydroxy-4-phenylcoumarin (**116**); 5-*O*-β-d-glucopyranosyl-7-methoxy-3′,4′-dihydroxy-4-phenylcoumarin (**109**); 6″-*O*-acetyl-5-*O*-β-d-galactopyranosyl-7,4′-dihydroxy-4-phenylcoumarin (**123**); 6″-*O*-acetyl-5-*O*-β-d-galactopyranosyl-7,3′,4′-trihydroxy-4-phenylcoumarin (**124**).	[56,59]
*Ibervillea sonorae* (S. Watson) Greene (Cucurbitaceae)/Roots.	1-Monopalmitin (**125**); glyceryl-1-monomargarate (**126**); 1-monostearin (**127**); glyceryl-1-monononadecylate (**128**); glyceryl-1-monoarachidate (**129**); glyceryl-1-monobehenate (**130**); glyceryl-1-monotricosanoate (**131**); glyceryl-1-monotetracosanoate (**132**); glyceryl-1-monopentacosanoate (**133**); glyceryl-1-monohexacosanoate (**134**); glyceryl-1-monooctacosanoate (**135**); lauric acid (**136**); myristic acid (**137**); pentadecanoic acid (**138**); palmitic acid (**139**); stearic acid (**140**); gallic acid (**141**).	[60,61,62,63,64]
*Ipomoea pes-caprae* (L.) R. Br and Ipomoea purga (Wender.) Hayne (Convolvulaceae)/Roots.	Pescapreins I (**142**), III (**143**), V (**144**) and IX (**145**); stolonoferins I (**146**) and III (**147**); murucoidins IV (**148**), V (**149),** XIV (**150**), XVIII (**151**), XIX (**152**) and XX (**153**); purginosides I (**154**), II (**155**) and IV (**156**); purgins I (**157**), II (**158**) and III (**159**); tricolorins A (**160**), E (**161**) and I (**162**); wolcottines I (**163**), I (**164**), II (**165**), III (**166**) and IV (**167**); intrapilosin VII (**168**).	[65]
*Justicia spicigera* Schltdl (Acanthaceae)/Leaves.	Kaempferitrin (**169**)	[66]
*Ligusticum porteri* J.M. Coult. & Rose (Apiaceae)/Roots.	(*Z*)-6,6′,7,3′α-Diligustilide (**170**); (*Z*)-ligustilide (**171**); 3-(*Z*)-butylidenephthalide (**172**); myristicin (**173**), Ferulic acid (**174**).	[67]
*Melampodium perfoliatum* (Cav.) Kunth (Asteraceae)/Aerial.	Perfoliatin A (**175**).	[68]
*Mosannona depressa* (Baill.) Chatrou (Annonaceae)/Roots.	2-Hydroxy-3,4,5-trimethoxy-1-(2′,4′-hydroxy-3′-dihydroxy) butylbenzene (**176**); 2-hydroxy-3,4,5-trimethoxy-1-(2′,3′,4′-hydroxy) butyl-benzene (**177**); 3-(3-hydroxy-2,4,5-trimethoxyphenyl) propane-1,2 diol (**178**).	[32,69,70,71]
*Opuntia streptacantha* Lem. (Cactaceae)/Cladodes.	4-Hydroxyphenylacetic acid (**179**).	[72,73]
*Psacalium decompositum* (A.Gray) H.Rob. & Brettell (Asteraceae)/Roots.	Cacalol (**180**); cacalol acetate (**181**); cacalone (**182**); maturin (**183**); maturinone (**184**).	[74,75,76,77]
*Psacalium paucicapitatum* (B.L.Rob. & Greenm.) H.Rob. & Brettell (Asteraceae)/Corms.	Kestose (**185**); nystose (**186**); fructofuranosyl-nystose (**187**).	[78]
*Phoradendron reichenbachianum* (Seem.) Oliv. (Santalaceae)/Leaves and stems.	Moronic acid (**188**); morolic acid (**189**); oleanolic acid (**70**); ursolic acid (**118**); 3,4-seco-olean-18-ene-3,28-dioic acid (**190**); α-amyrin (**191**); β-amyrin (**192**); oleanolic aldehyde (**193**); lupeol (**72**); lupenone (**194**); betulin aldehyde (**195**); betulon aldehyde (**196**); betulinic acid (**73**); acacetin (**21**); betulonic acid (**197**); squalene (**198**); triacontanol (**199**); β-sitosteryl linoleate (**200**); stigmasteryl linoleate (**201**); β-sitosterol (**34**); stigmasterol (**33**); acacetin 7-methyl ether (**202**).	[79,80,81]
*Rhizophora* mangle L. (Rizophoraceae)/Cortex.	Cinchonains Ia (**203**) and Ib (**204**); epicatechin (**113**); catechin-3-*O*-rhamnopyranoside (**205**); lyoniside (**206**); nudiposide (**207**).	[19,82,83,84]
*Salvia circinnata* Cav. (Lamiaceae)/Aerial.	6,6″,3″-Trihydroxy-7,3′,7′-*O*-trimethyl-loniflavon (**208**); amarisolides B (**209**), C (**210**), D (**211**) and E (**212**); amarisolide (**213**); pedalitin (**214**); apigenin-7-*O*-β-d-glucopyranoside (**215**); 2-(3,4-dimethoxy-phenyl)-5,6-dihydroxy-7-methoxy-*4H*-chromen-4-one (**216**).	[85]
*Smilax aristolochiifolia* Mill. (Smilaceae)/Roots.	*N*-*trans*-Feruloyltyramine (**217**); astilbin (**218**); chlorogenic acid (**13**).	[86,87]
*Smilax moranensis* M. Martens & Galeotti (Smilaceae)/Roots.	*trans*-Resveratrol (**219**); 5-*O*-caffeoylquinic acid (**220**); chlorogenic acid (**13**).	[19,84,88,89]
*Swietenia humilis* Zucc (Melaiceae)/Seeds.	2-Hydroxy-destigloyl-6-deoxyswietenine acetate (**221**)^;^ humulin B (**222**); methyl-2-hydroxy-3-β-isobutyroxy-1-oxomeliac-8(30)-enate (**223**); methyl-2-hydroxy-3-β-tigloyloxy-1-oxomeliac-8(30)-enate (**224**); humilinolide G (**225**); humilinolide C (**226**); methyl-2-hydroxy-3-β-isobutyloyl-8α,30α-epoxy-1-oxo-meliacate (**227**); humilinolide H (**228**).	[90,91,92]
*Tecoma stans* (L.) Juss. ex Kunth (Bignoniaceae)/Leaves.	Chrysoeriol (**229**); apigenin (**230**); luteolin (**231**); verbascoside (**232**); luteolin-7-*O*-glucopyranoside (**233**).	[93,94]
*Turnera diffusa* Willd. ex Schult. (Passifloraceae)/Aerial.	Teuhetenone A (**234**).	[95]

**Table 2:** Activity Found in the Isolated Compounds from Mexican Plants.

Plant/Extract	Experiment ^1^	AC ^2^	Other Activity Found
*Acacia angustissima*/Methanol extract (**ME**).	In vivo: **ME**: AHT (25, 50, and 100 mg/kg of bw) and OGTT (25, 50, and 100 mg/kg of bw) in healthy and STZ-treated rats. BP: TC, TG, LDL and HDL [12]. In vitro: Lipid peroxidation and protein content in kidney, glucose incorporation assay in adipocytic cells.	**1** **2** **3**	**1** Decreases blood glucose in diabetic rats; insulin-sensitizing [96,97]. **2** is in vitro and in vivo AG inhibitor [98], and hypoglycemic (CHT), glucose oxidizing and insulin-mimetic agent [99]. **3** lowers blood glucose level (CHT), glucose-6-phosphatase, and fructose-1,6-bisphosphatase; increases the activities of hexokinase, G6PD, and GSH by increasing level of insulin; reduces the total cholesterol and triglycerides in both plasma and tissues i.e., liver and kidney [100].
*Acourtia thurberi/*Aque-ous extract (**WE**).	In vivo: **WE**: AHT, OGTT, and OSTT in healthy and STZ-treated mice using half-log interval doses (31.6, 100, and 316.2 mg/kg of bw of the extract and 3.2, 10, and 31.6 mg/kg of bw of compounds for all experiments) [13]. In vitro: **WE**: Y-AG (IC_50_ = 566.7 μg/mL).	**4** ^(31.6 mg/kg)^ **5** ^(3.2-31.6 mg/kg/944.9 μM)^ **6** ^(3.2-31.6 mg/kg/944.9 μM)^ **7** ^(3.2-31.6 mg/kg/3.98 μM)^	-
*Acosmium panamense/*Aq-eous (**WE**) and butanol (**BE**) extracts.	In vivo: **WE**: AHT (20 and 200 mg/kg of bw) in STZ-treated rats. **BE**: AHT (20 and 100 mg/kg of bw) STZ-treated rats. Dose of compounds **9** and **10** for AHT: 20 mg/kg of bw [15].	**9** ^(20 mg/kg)^ **10** ^(20 mg/kg)^	-
*Agarista mexicana/*Chlo-roform extract (**CHE**).	In vivo: **CHE**: AHT in healthy (150 mg/kg of bw) and alloxan-treated mice (50, 100 and 150 mg/kg of bw) and OGTT in alloxan-treated rats (150 mg/kg of bw). Dose of compounds **11** and **12** for AHT was 50 mg/kg of bw [17].	**11** ^(50 mg/kg)^ **12** ^(50 mg/kg)^	-
*Ageratina petiolaris/*Aqu- eous (**WE**) and methanol (**ME**).	In vivo: **WE**: AHT (40 and 160 mg/kg of bw), OGTT (160 mg/kg bw); PTT (160 mg/kg of bw) in STZ-NA-treated rats. **ME**: AHT (67 and 268 mg/kg of bw) in STZ-NA-treated rats. Dose of **14** for AHT was 3.73 mg/kg of bw [18]. In vitro: **WE**: G6Pase Activity (IC_50_ = 223 μg/mL) [19].	**13** ^(56 μg/mL)^ **14** ^(3.73 mg/kg)^	-
*Annona cherimola/*Etha-nol extract (**EE**).	In vivo: **EE**: AHT, CHT in healthy and alloxan-treated rats; OGTT and OSTT in Normoglycemic rats at a dose of 300 mg/kg of bw for the extract and 30 mg/kg of bw for **20** in all experiments (AHT, CHT, OGTT and OSTT); **20** was active in all experiments [20].	**20** ^(30 mg/kg)^	-
*Anoda cristata/*Mucil-age (**M**), free mucilage aqueous (**FM-WE**), aqueous (**WE**) and organic (**OE**) extracts.	In vivo: **WE** and **M** tested in AHT, OGTT, and OSTT in healthy and STZ-NA-treated mice (31.6, 100, and 316 mg/kg of bw). **FM-WE**: AHT, OGTT, and OSTT in healthy and STZ-NA-treated mice (31.6, 100, and 316 mg/kg of bw); OGTT and CHT in metabolic syndrome induced rats (100, and 316 mg/kg of bw). BP: cholesterol, TG, uric acid and glucose. (most active). **OE**: OSTT in healthy and STZ-NA-treated mice (31.6, 56.2, and 100 mg/kg of bw). Doses of **21** and **22** for AHT were 3, 10, and 31.6 mg/kg of bw [21].	**21** ^(3, and 31.6 mg/kg)^ **22^(^** ^3-31.6 mg/kg)^	**21** and **22** are PPAR agonists and antioxidants [101].
*Artemisia ludoviciana/*Es- sential oil (**EO**), organic (**OE**) and aqueous (**WE**) extracts.	In vivo: **EO**, **OE**, and **WE** tested in AHT, OGTT, and OSTT in healthy and STZ-treated mice (31.6, 100, and 316 mg/kg of bw). Isolated compounds: Cotreatment with Ca2^+^ and K^+^ ion channels regulators (17.7 mg/kg bw); the doses of **23** and **25** for AHT were 5.6, 17.7, and 31.6 mg/kg of bw. In vitro: Y-AG for isolated compounds [22].	**23** ^(17.7, 31.6 mg/kg; 0.49 μM)^ **24** **25** ^(5.6,17.7 and 31.6 mg/kg)^ **26** ^(545.2 μM)^ **27**	**23** and **24** lowers blood glucose levels through the up-regulation of GK activity, plasma insulin and adiponectin concentration, downregulated G6Pase and PEPCK activities, and sustained pancreatic β-cell function [102,103]. **27**: inhibits AG [104].
*Arracacia tolucensis/*Hex-ane (**HE**), ethyl acetate (**EAE**) and ethanol (**EE**) extracts.	In vivo: **HE, EAE**, **EE**: CHT (250 mg/kg of bw). Hematic biometry and BP: urea, creatinine, cholesterol, TG, HDL, LDL, VLDL, AST, ALT, and bilirubin; **EAE** was the most active) [23].	no compounds were tested in this experiment	-
*Brickellia veronicaefolia/* Essential oil (**EO**), chloroform (**CHE**) and organic (**OE**) extracts.	In vivo: **OE** and **EO**: AHT, OGTT in healthy and STZ-NA-treated mice (**OE** doses: 30, 100, and 300 mg/kg of bw; **EO** doses: 10 mg/kg of bw). **CHE**: isolation of compounds for testing in AHT in healthy and alloxan-treated mice. The doses of **30** for AHT were 10, 25, and 50 mg/kg of bw [24].	**30** ^(50 mg/kg)^	-
*Bromelia karatas/*Ethanol:water (**EWE**), aqueous (**WE**) and organic (**OE**) extracts.	In vivo: **WE** tested in AHT (35 and 350 mg/kg of bw), CHT (218 mg/kg of bw) and PTT (218 mg/kg) in STZ-NA rats. **EWE** in AHT (30 and 350 mg/kg of bw) in STZ-NA rats. BP: HbA1c, HDL, TG and cholesterol. The doses of **31**, **3** and **32** for AHT were 72, 3.63, and 1.8 mg/kg of bw, respectively [26,27]. In vitro: G6Pase Activity [19].	**31** ^(72 mg/kg)^ **32** ^(1.8 mg/kg)^ **3** ^(3.63 mg/kg)^ **33**	**31** and **33** have hypoglycemic effects in STZ-NA rats treated with doses of 0.25 and 0.50 mg/kg for 21 days to improve biochemical and hematological parameters [105].
*Calea oliveri/*Aqueo-us extract (**WE**) and essential oil (**EO**).	In vivo: **WE** tested in AHT, OGTT, and OSTT in healthy and STZ-NA mice (dose of 56, 100, and 316 mg/kg of bw for all experiments). **EO**: OSTT (31.6, 100 and 316 mg/kg of bw). The dose of **39** for OSTT were 5.6, 10, and 31.6 mg/kg of bw; the dose of both **42** and **43** for OSTT were 3.16, 7 and 10 mg/kg of bw. In vitro: Y-AG for **WE** (IC_50_ = 0.169 mg/mL) and isolated compounds [28,29].	**21** **27** **39** ^(5.6-31.6 mg/kg)^ **40** ^(0.42 mM)^ **41** **42** ^(3.16-10 mg/kg)^ **43** ^(3.16-10 mg/kg; 0.28 mM)^ **45** ^(0.16 mM)^ **46** **47** ^(0.53 mM)^	**46** Restores PA-induced loss of β-cell mass and function through AMPK/mTOR-mediated autophagy [106]; inhibits AG [107]. **45** and **47** Increase glucose uptakes in skeletal muscle by activating the JAK/STAT pathway, and by CaMKKβ/AMPK and insulin signalling pathways, respectively [108].
*Cecropia obtusifolia/*But-anol (**BE**) and aqueous (**WE**) extracts.	In vivo: **WE** tested in AHT (90 and 150 mg/kg of bw) in STZ-treated rats. CHT in diagnosed type 2 diabetic patients. BP: Serum glucose, cholesterol, TG and insulin levels were determined every 15 days; HbA1c, ALT, AST, and ALKP measured every month. **BE**: AHT (9 and 15 mg/kg of bw), OMTT (96 mg/kg of bw) in STZ-NA-treated rats. The dose of both **13** and **54** for AHT were 10 mg/kg of bw). In vitro: Y-AG for **BE** (IC_50_ =14 μg/mL); adipogenesis and 2-NBDglucose uptake in 3T3-F442A murine adipocytes [30,31,32].	**13** ^(10 mg/kg)^ **54** ^(10 mg/kg)^	**54** Inhibits AG [109].
*Cochlospermum vitifolium/*Hex- ane (**HE**), dichlorometh-ane (**DE**) and methanol (**ME**) extracts.	In vivo: **HE** and DE assay in AHT (120 mg/kg of bw) in healthy and STZ-NA-treated rats. **ME**: in AHT (100 mg/kg of bw), OGTT (100 mg/kg of bw), CHT (100 mg/kg of bw) in healthy and STZ-NA-treated rats. BP: Glucose, total cholesterol, HDL and TG. In vitro: Hepatoprotective activity assay and RI-AG for **ME** (IC_50_ = 1.9 mg/ML) [34,35].	**55**	**55** Could prevent functional changes in vascular reactivity in diabetic rats through nitric oxide- and no prostaglandin-dependent pathways [110].
*Coriandrum sativum/*Aque-ous extract (**WE**).	In vivo: **WE** tested in OSTT (100, 300, and 500 mg/kg of bw) in healthy rats. The dose of **20** for OSTT was 50 mg/kg of bw). In vitro: Y-AG for **WE** (IC_50_ = 1.63 mg/mL) [36].	**20** ^(50 mg/kg)^	-
*Cucurbita ficifolia/*Juice (**J**) and aqueous (**WE**) extracts.	In vivo: **J** tested in AHT (4ml/kg) in Type 2 diabetic patients with moderate hyperglycemia; AHT (125, 250, 500, 594.49, 750, 1000, and 1250 mg/kg of bw) and CHT (1000 mg/kg of bw) in healthy and alloxan-treated mice. **EW**: CHT (200 mg/kg of bw) in STZ-treated mice [37,39,41]. In vitro: Effect on [Ca2^+^]i in RINm5F cells. Viability assays using DRAQ7™ probe. Participation of *C. ficifolia* as regulator of [Ca2^+^]i through K^+^ ATP channels [40].	**3**	-
*Equisetum myriochaetum/*Aqueous (**WE**) and butanol (**BE**) extracts.	In vivo: **WE** and **BE** assayed in AHT (7 and 13, 8 and 16 mg/kg of bw for **WE** and **BE,** respectively) in STZ-treated rats. **WE** tested in AHT (330 mg/kg of bw) in type 2 diabetic patients. BP: Glucose, TG, cholesterol, and glycated hemoglobin. OMTT (96 mg/kg of bw) and PTT (330 mg/kg of bw) in STZ-treated rats. Dose not reported for **62** in AHT. In vitro: G6Pase activity and Y-AG for **WE** [19,32,43,44].	**62**	-
*Eysenhardtia platycarpa/*Met-hanol extract (**ME**).	In vivo: **ME** tested in AHT (30, 100, and 300 mg/kg of bw) in STZ-treated rats. The doses of **69** for AHT were 3.1, 10, and 31 mg/kg of bw [45,46].	**69** ^(31 mg/kg)^ **2** **70** **34**	**70** Acts as hypoglycemic and anti-obesity agent mainly through reducing the absorption of glucose, decreasing endogenous glucose production, increasing insulin sensitivity, improving lipid homeostasis, and weight regulation [111].
*Eysenhardtia polystachya/*A-queous (**WE**) and methanol: water (**MWE**) extracts.	In vivo: **WE** tested in AHT in alloxan-treated mice. **MWE** in AHT (100, 200, and 400 mg/kg of bw) in STZ-treated mice; CHT (400 mg/kg of bw) in STZ-treated mice; OGTT (400 mg/kg of bw) in normal and STZ-treated mice. Compound **104**: Tested in experimental diabetic nephropathy model to study pathological changes in the kidney (dose: 100 mg/kg of bw) [47,48,49,50,51]. In vitro: **MWE** tested for determining advanced glycation end-product formation [50].	**93** **96** **99** **102** **104** ^(100 mg/kg)^	-
*Exostema caribaeum*/Aq-ueous extract (**WE**).	In vivo: **WE** tested in AHT and OSTT in healthy and STZ-NA-treated mice. Doses of 100, 300, and 500 mg/kg of bw for all experiments [52].	**13** **106**	-
*Hamelia patens/*Ethanol:water (1:1) (**EWE**), aqueous (**WE**) and methanol (**ME**) extracts.	In vivo: **EWE** and **WE** tested in AHT (30 and 300 mg/kg of bw, and 60 and 600 mg/kg of bw for **EWE** and **WE,** respectively) in STZ-NA-treated rats. **ME** assayed **in** CHT (35, 75 and 150 mg/kg of bw) in healthy and STZ-treated rats [53,54]. In vitro: Y-AG for **ME** (IC_50_ = 78.3 μg/mL).	**13** **47** **2** **112** **113**	**112** Exhibits significant potential as an antidiabetic agent by suppressing the progression of type 2 diabetic states that is suggested by attenuation of hepatic glucose output and enhancement of adipocyte glucose uptake, insulin secretion, and antioxidant capacity [112]. **113** improves insulin sensitivity in high fat diet-fed mice and inhibits AG [113,114].
*Hintonia latiflora/*Orga- nic (**OE**) and aqueous (**WE**) and endophytic fungus extracts.	In vivo: **OE** tested in AHT (10, 30, 100, and 300 mg/kg of bw) in healthy and STZ-treated rats. In CHT (50 and 100 mg/kg of bw) in STZ rats. The doses of compounds **106**-**109** and **114**-**117** for CHT were 15 and 30 mg/kg of bw. **WE** tested in AHT (100, 300 and 500 mg/kg of bw), OSTT (100, 300, and 500 mg/kg of bw) in healthy and STZ-NA-treated rats. The doses of **122** for AHT and OSTT were 3.1, 10, and 31.6 mg/kg of bw [55,56,57,58,115]. The doses of **116** for OSTT was 50 mg/kg of bw. In vitro: Determination of hepatic glycogen, Y-AG for compounds.	**106** ^(30 mg/kg)^ **107** ^(30 mg/kg)^ **108** ^(30 mg/kg)^ **109** ^(30 mg/kg)^ **115** ^(30 mg/kg)^ **116** ^(30 mg/kg)^ **120** ^(23.8 μM)^ **121** ^(15.8 μM)^ **122** ^(AHT: 31.6mg/kg; OSTT:10 mg/kg; 22.1 μM)^ **13** **118**	-
*Hintonia standleyana*/Or-ganic extract (**OE**).	In vivo: **OE** tested in AHT (10 and 100 mg/kg of bw) in healthy and STZ-treated rats; CHT (50 and 100 mg/kg of bw) in STZ rats and developing hyperglycemic situation in rats. The doses of compounds **115, 116, 123**, and **124** for AHT were 10 mg/kg of bw. The doses of both **115** and **116** for CHT were 15 and 30 mg/kg of bw [56,59].	**115** ^(15 mg/kg)^ **116** ^(15 mg/kg)^ **123** ^(10 mg/kg)^ **124** ^(10 mg/kg)^ **109**	-
*Ibervillea sonorae/*Aque-ous (**WE**), juice (**J**), Dichlorometh-ane (**DE**) and methanol (**ME**) extracts.	In vivo: extracts tested in AHT in healthy and alloxan-treated mice (*ip* administration; the doses of **WE** were 150, 300, 600, and 850mg/kg of bw; dose for **J**, **DE**, and **ME**: 300 and was 600 mg/kg of bw). **WE**: Tested in a murine model of obesity and hyperglycemia, induced by a high-calorie diet; the relationship of these effects with hepatic oxidation were observed. In vitro: **WE** was assayed for glucose uptake in insulin- sensitive, and insulin-resistant murine and human cultured adipocytes; both in the absence or the presence of insulin signaling pathway inhibitors, and on murine and human adipogenesis [60,61,62,63,64].	-	-
*Ipomoea pes-caprae*/hexane (**HE**) and chloroform (**CHE**) extracts.	In vitro: Y-AG of isolated compounds [65].	**142** ^(626 μM)^ **144** ^(724 μM)^ **155** ^(1067 μM)^ **159** ^(330 μM)^	-
*Justicia spicigera/*Etha- nol extract (**EE**).	In vivo: **EE** tested in OSTT (100 mg/kg of bw) in healthy and STZ-NA-treated rats. Effect on the glucose uptake in insulin-sensitive and insulin-resistant murine 3T3-F442A and human subcutaneous adipocytes [66].	**169**	**169** Induces hypoglycemic effect in normal and in alloxan-induced diabetic rats; inhibits GLUT4 mediated glucose uptake in differentiated 3T3-L1 cells by interfering with the insulin signaling pathway, and by directly interacting with membrane GLUT4 [116,117].
*Ligusticum porteri/*Organic extract (**OE**).	In vivo: **OE** tested in AHT, OGTT, and OSTT in healthy and STZ-NA mice; the doses were 56.2, 100, and 316 mg/kg of bw for all experiments. The doses of **170**–**171** for OGTT were 10, 31.2 and 56.2 mg/kg of bw for all compounds. The doses of **172** for OSTT were 10 and 56.2 mg/kg of bw. In vitro: Y-AG for isolated compounds [67].	**171** ^(10-56.2 mg/kg)^ **172** ^(10 and 56.2 mg/kg; 2.5 mM)^	-
*Melampodium perfoliatum/*Aq-ueous extract (**WE**).	In vivo: OSTT in STZ-NA-treated mice for isolated compound **175** (doses: 3.16, 10 and 31.6 mg/kg of bw). In vitro: RI-AG for extract (IC_50_ = 985.2 µg/mL) and isolated compound [68].	**175** ^(3.16-31.6 mg/kg; 6.5 mM)^	-
*Mosannona depressa*/Aqu-eous (**WE**), butanol (**BE**) and ethanol (**EE**) extracts.	In vivo: AHT in STZ-treated rats for **WE** (40 and 80 mg/kg of bw), **EE** (113 mg/kg of bw) and **BE** (80 mg/kg bw); the last one was the most active. **BE** tested in OMTT (96 mg/kg of bw) and CHT (50 mg/kg of bw) in STZ-treated rats; and stimulation of insulin secretion in STZ-treated rats; BP measuring glucose, TG, cholesterol, and glycosylated hemoglobin were measured. **EE**: PTT (60 and 80 mg/kg of bw) in n5-STZ rats after an 18-h fasting period. In vitro: Effect on glucose-6-phosphatase activity for **EE** (IC_50_= 267.62 μg/mL) and Y-AG for **BE** (IC_50_= 267.62 μg/mL) [32,69,70,71].	-	-
*Opuntia streptacantha/*Li-quefied (**LE**) filtrate extract (**FE**) and juice (**J**).	In vivo: **LE** tested in AHT (135 mg/kg of bw) and MTT (135 mg/kg of bw) in n5-STZ rats. **FE**: in AHT (12 and 27 mg/kg of bw) and MTT (12 and 27 mg/kg of bw) in n5-STZ rats. **J** in MTT (4 mL/kg) in n5-STZ rats. In vitro: RI-AG [72,73].	-	-
*Psacalium decompositum/*Aqueous (**WE**), methanol (**ME**) and hexane (**HE**) extracts.	In vivo: **WE** tested in AHT (50, 100, 200, or 400 mg/kg of bw) in healthy and alloxan mice; in OGTT (dose not specified) in healthy rabbits; CHT (150 mg/kg of bw) in rats with 12 weeks fructose feeding. **ME** and **HE** tested in AHT (50, 100, 200, or 400 mg/kg of bw for both extracts) in healthy mice. The doses of **180**-**183** for AHT were 50 and 100 mg/kg of bw [74,75,76,77]. In vitro: Compounds tested in diazoxide-induced relaxation of male rat aortic rings precontracted with phenylephrine.	**180** **182**	-
*Psacalium paucicapitatum* Aqueous extract (**WE**).	In vivo: **WE** tested in CHT and OGTT in mice with 12 weeks fructose feedings [78].	-	-
*Phoradendron reichenbachianum/*Acetone extract (**AE**)	In vivo: **AE** tested in AHT (100 mg/kg of bw) in STZ-NA rats. CHT, OGTT, and OSTT for isolated compounds in STZ-NA rats (the doses of all the compounds tested were 50 mg/kg of bw) [79,80,81]. In vitro: Inhibitory activity of compounds against protein tyrosine phosphatase1B (PTP-1B). Assay for 11β-HSD1 inhibition [79].	**188** ^(50 mg/kg)^ **189** ^(50 mg/kg)^ **70** ^(50 mg/kg)^ **118** ^(50 mg/kg)^ **21** **34**	**34** Attenuates insulin resistance in adipose tissue via IRS-1/Akt mediated insulin signaling in high fat diet and sucrose induced type-2 diabetic rats [118].
*Rhizophora mangle/*Aque-ous (**WE**) and ethanol:water (**EW**) extracts.	In vivo: **WE** tested in AHT (5.9 and 59 mg/kg of bw), OMTT (56 mg/kg of bw) in STZ-NA-treated rats. **EW** assayed in AHT (9 and 90 mg/kg of bw), CHT (90 mg/kg of bw) and PTT (90 mg/kg) in healthy and STZ-NA rats [19,82,83,84]. In vitro: G6Pase activity for **EW** (IC_50_= 99 μg/mL) and RI-AG [19,32].	**113** **204**	**204** Induces insulin secretion in vitro and in vivo [119].
*Salvia circinnata/*Aqu-eous extract (**WE**).	In vivo: **WE** tested in AHT, OGTT, and OSTT in healthy and STZ-NA-treated mice. Doses of 31.6, 100 and 316 mg/kg of bw for all experiments. The doses for **213** and **214** for OSTT were 3.1, 10, and 31.6 mg/kg of bw, and 1, 3.1, and 10 mg/kg of bw, respectively. In vitro: RI-AG [85].	**208** ^(39 μM)^ **213** ^(3.1-31.6 mg/kg; 500 μM)^ **214** ^(1-10 mg/kg; 810 μM)^ **215** ^(200 μM)^ **216** ^(1800 μM)^	-
*Smilax aristolochiifolia/*Acetone (**AE**), ethanol:water (**EWE**) and aqueous (**WE**) extracts.	In vivo: **AE** and **217** (25 mg/kg of bw) tested in the insulin tolerance curve in mice with a high-caloric diet. In vitro: Pancreatic α-amylase and Y-AG testing for **WE**, **EWE,** and compounds [86,87].	**217** ^(25 mg/kg)^ **13**	-
*Smilax moranensis/*Aq-ueous (**WE**) and ethanol (**EE**) extracts.	In vivo: **WE** tested in AHT (20 and 200 mg/kg of bw) in n5-STZ-treated rats. **EE** assayed in AHT (8 and 80 mg/kg of bw), CHT (80 mg/kg of bw), PTT (80 mg/kg of bw), MTT (80 mg/kg of bw) in healthy and STZ-NA rats; and BP measuring glycated hemoglobin (HbA1c) and lipid profile (HDL, TG and cholesterol). In vitro: G6Pase activity for EE (IC_50_ = 84 μg/mL) and Y-AG [19,84,88,89].	**13** ^(63 μg/mL)^ **219**	**219** Induces effects that might contribute to the protection of β cells in diabetes; it reduces insulin secretion in animals with hyperinsulinemia [120,121].
*Swietenia humilis/*Aqueo-us extract (**WE**).	In vivo: **WE** tested in AHT (31.6, 100, and 316 mg/kg of bw) OGTT (31.6, 100, and 316 mg/kg of bw), OSTT (100, 177, and 316 mg/kg of bw) in healthy and STZ-NA-treated mice; OGTT (100 and 316 mg/kg of bw) in metabolic syndrome in Sprague Dawley rats (FF-MS). CHT (100 and 316 mg/kg of bw) in FF-MS-induced rats; BP measuring glucose, TG, total cholesterol and uric acid. The doses of all **221**, **222**, and **224** for AHT and OGTT were 3.16, 10 and 31.6 mg/kg of bw. In vitro: Measurement of hepatic glycogen content and serum insulin levels. Studies on INSE1, H4IIE and C2C12 cells to assess insulin secretion; glucose uptake and mitochondrial bioenergetics, respectively; and glucose-6-phosphatase inhibition [90,91,92].	**221** ^(3.16-31.6 mg/kg)^ **222** ^(3.16-31.6 mg/kg)^ **224** ^(3.16-31.6 mg/kg)^ **228** ^(16.27 μM)^	-
*Tecoma stans/*Aqueous (**WE**) and ethanol:water (**EWE**) extracts.	In vivo: **WE** tested in AHT (500 mg/kg of bw), CHT (125, 250, and 500 mg/kg of bw), OGTT (500 mg/kg of bw) and OSTT (125, 250, and 500 mg/kg of bw) in healthy and STZ-treated rats. In vitro: Pancreatic lipase inhibition for **EWE** (30% inhibition) and compounds [93,94].	**229** ^(85.03%)^ **231** ^(32.83%)^ **232** ^(36.29%)^	**231** Inhibits alpha glucosidases [109].
*Turnera diffusa/*Metha-nol extract (**ME**).	In vivo: **ME** assayed in AHT in normoglycemic and alloxan-treated mice. The doses of **234** for AHT were 1 and 5 mg/kg of bw. In vitro: Y-AG [95].	**234** ^(1-5mg/kg;^ ^> 330μg/mL)^	-

^1^**AHT:** Acute hypoglycemic test. **OGTT:** Oral glucose tolerance test. **OSTT:** Oral sucrose tolerance test. **OMTT:** Oral maltose tolerance test. **CHT:** Chronic hypoglycemic test. **Y-AG:** Yeast α-glucosidase inhibition. **RI-AG:** Rat intestinal α-glucosidase inhibition. **BP:** Blood Biochemical profile. **PTT:** Pyruvate tolerance test. ^2^ Active compound with defined mechanism of action: **α-glucosidase;** increasing plasmatic insulin levels; insulin sensitivity; other mechanisms; unknown mechanism of action, (active dose/IC_50_).
